# CXCR2 antagonist navarixin in combination with pembrolizumab in select advanced solid tumors: a phase 2 randomized trial

**DOI:** 10.1007/s10637-023-01410-2

**Published:** 2024-02-07

**Authors:** Andrew J. Armstrong, Ravit Geva, Hyun Cheol Chung, Charlotte Lemech, Wilson H. Miller Jr., Aaron R. Hansen, Jong-Seok Lee, Frank Tsai, Benjamin J. Solomon, Tae Min Kim, Christian Rolfo, Vincent Giranda, Yixin Ren, Fang Liu, Bhargava Kandala, Tomoko Freshwater, Judy S. Wang

**Affiliations:** 1 Duke Cancer Institute Center for Prostate and Urologic Cancers, Duke University, Durham, NC 27710 USA; 2 Division of Oncology, Tel Aviv Sourasky Medical Center, Tel Aviv, Israel, affiliated to the Sackler School of Medicine, Tel Aviv University, Tel Aviv, Israel; 3 Yonsei Cancer Center, Yonsei University Health System, Seoul, South Korea; 4 Scientia Clinical Research, Randwick, NSW Australia; 5 Segal Cancer Center, McGill University, Jewish General Hospital, Montreal, QC Canada; 6 Princess Margaret Cancer Centre, Toronto, ON Canada; 7 Seoul National University Bundang Hospital, Gyeonggi-do, South Korea; 8 Honor Health, Scottsdale, AZ USA; 9 Peter MacCallum Cancer Centre, Melbourne, VIC Australia; 10 Seoul National University Hospital, Seoul, South Korea; 11 Center for Thoracic Oncology, Icahn School of Medicine at Mount Sinai, The Tisch Cancer Institute, New York, NY USA; 12 Merck & Co., Inc, Rahway, NJ USA; 13 Florida Cancer Specialists/Sarah Cannon Research Institute, Sarasota, FL USA

**Keywords:** Clinical trial, C-X-C chemokine, Navarixin, Pembrolizumab, Solid tumors

## Abstract

C-X-C motif chemokine receptor 2 (CXCR2) has a role in tumor progression, lineage plasticity, and reduction of immune checkpoint inhibitor efficacy. Preclinical evidence suggests potential benefit of CXCR2 inhibition in multiple solid tumors. In this phase 2 study (NCT03473925), adults with previously treated advanced or metastatic castration-resistant prostate cancer (CRPC), microsatellite-stable colorectal cancer (MSS CRC), or non–small-cell lung cancer (NSCLC) were randomized 1:1 to the CXCR2 antagonist navarixin 30 or 100 mg orally once daily plus pembrolizumab 200 mg intravenously every 3 weeks up to 35 cycles. Primary endpoints were investigator-assessed objective response rate (RECIST v1.1) and safety. Of 105 patients (CRPC, n=40; MSS CRC, n=40; NSCLC, n=25), 3 had a partial response (2 CRPC, 1 MSS CRC) for ORRs of 5%, 2.5%, and 0%, respectively. Median progression-free survival was 1.8–2.4 months without evidence of a dose-response relationship, and the study was closed at a prespecified interim analysis for lack of efficacy. Dose-limiting toxicities occurred in 2/48 patients (4%) receiving navarixin 30 mg and 3/48 (6%) receiving navarixin 100 mg; events included grade 4 neutropenia and grade 3 transaminase elevation, hepatitis, and pneumonitis. Treatment-related adverse events occurred in 70/105 patients (67%) and led to treatment discontinuation in 7/105 (7%). Maximal reductions from baseline in absolute neutrophil count were 44.5%−48.2% (cycle 1) and 37.5%−44.2% (cycle 2) and occurred within 6−12 hours postdose in both groups. Navarixin plus pembrolizumab did not demonstrate sufficient efficacy in this study. Safety and tolerability of the combination were manageable. (*Trial registration*: ClinicalTrials.gov, NCT03473925).

**Supplementary Information:**

The online version contains supplementary material available at 10.1007/s10637-023-01410-2.

## Introduction

C-X-C motif chemokine receptor 2 (CXCR2) signalling plays an important role in inflammatory diseases and cancer [1]. CXCR2 inhibits proliferation in normal cells, whereas its presence in the tumor microenvironment is associated with increased tumor cell proliferation [1, 2]. CXCR2 expression is upregulated in a number of tumor types, including prostate cancer, colorectal cancer (CRC), and non–small-cell lung cancer (NSCLC) [3–5], and expression of CXCR2 and its ligands is associated with poor patient prognosis [4, 5]. CXCR2 expression has been implicated in prostate cancer lineage plasticity and neuroendocrine transformation in the setting of resistance to potent androgen receptor inhibition and is normally expressed on tissue resident neuroendocrine cells [6]. Increased CXCR2 expression in tumor cells supports cell survival, epithelial-mesenchymal transition, and recruitment of myeloid-derived suppressor cells (MDSC) to the tumor microenvironment, which promote tumor progression and protect tumors from the body’s natural antitumor immune response [1, 3, 7, 8]. Additionally, high levels of circulating MDSC in the tumor microenvironment may induce resistance to immune checkpoint inhibitors in tumor types responsive to these agents [9].

Navarixin (formerly SCH-527123, MK-7123) is an orally available small-molecule CXCR2 antagonist. In patients with chronic obstructive pulmonary disease [10] and asthma [11], navarixin demonstrated reductions in absolute neutrophil count (ANC) as well as sputum neutrophil count, a marker associated with airway inflammation, that corresponded with improvements in forced expiratory volume in 1 second, suggesting clinical benefits [12]. Results of preclinical studies suggested potential therapeutic relevance in different cancers [6, 13–17]. For example, navarixin reversed neuroendocrine lineage plasticity and enzalutamide resistance in metastatic castration-resistant prostate cancer (CRPC) preclinical models [6], inhibited tumor growth in CRC and melanoma *in vivo* [13, 14], inhibited colony formation of triple-negative breast cancer and pancreatic ductal adenocarcinoma cells [15, 16], and induced apoptosis of malignant cells in liver metastases *in vivo* [17]. Given the immunosuppressive role of CXCR2-recruited MDSC in the tumor microenvironment, CXCR2 antagonists like navarixin might complement the action of programmed cell death protein 1 (PD-1) or programmed cell death ligand 1 (PD-L1) inhibitors in tumor types with low response rates to these agents. Preclinical evidence suggests that combining CXCR antagonists with PD-1 inhibitors may enhance antitumor activity [18–20]. This phase 2 proof-of-concept study assessed the combination of 2 pharmacodynamic doses of navarixin plus pembrolizumab in 3 distinct cohorts of patients with advanced or metastatic CRPC, microsatellite-stable CRC (MSS CRC), and PD-(L)1–refractory NSCLC who were not predicted to benefit from PD-1 blockade alone. We were able to initiate this phase 2 study without a prior phase 1 study in patients with cancer because of the clinical experience with navarixin in healthy volunteers and patients with chronic obstructive pulmonary disease and asthma. These prior studies demonstrated expected treatment-induced CXCR2-specific pharmacodynamic biomarkers, including reduced peripheral blood- and organ-specific myeloid cells such as neutrophils accompanied by reductions in inflammatory biomarkers, and improvements in lung function without an increased risk of infectious complications [10, 11].

## Methods

All study procedures were in accordance with local and/or national regulations and the Declaration of Helsinki. The study protocol (MK-7123-034) was approved by the institutional review board or independent ethics committee at each site. Informed consent was obtained from all patients.

### Patients

Eligible patients were ≥18 years old and had histologically or cytologically confirmed unresectable stage III or IV CRPC (adenocarcinoma), locally advanced unresectable stage III or IV MSS CRC, or stage IV NSCLC with progression on, intolerance to, or ineligibility for all other treatments known to confer benefit. Patients with CRPC must have progressed on ≥1 second-generation antiandrogen therapy and have ongoing androgen deprivation with serum testosterone <50 ng/dL (<2.0 nM). Patients with CRPC receiving luteinizing hormone–releasing hormone agonists or antagonists must have initiated those treatments ≥4 weeks before the first dose of study drug and continued the treatment throughout the study. Patients with MSS CRC must have previously received standard treatments, including fluoropyrimidine, oxaliplatin, and irinotecan. Prior taxane chemotherapy was permitted but no prior treatment with PD-1 or PD-L1 inhibitors was allowed for patients with CRPC or MSS CRC. Patients with NSCLC must have had a PD-L1 tumor proportion score ≥50%, had no sensitizing *EGFR* mutations or *ALK* translocations amenable to tyrosine kinase inhibitor therapy, and disease must have progressed on prior monotherapy or combination therapy with an anti–PD-(L)1 antibody. All patients were required to have measurable disease by Response Evaluation Criteria in Solid Tumors version 1.1 (RECIST v1.1), Eastern Cooperative Oncology Group (ECOG) performance status of 0 or 1, and adequate organ function.

### Study design

This was an open-label, parallel-group, multicenter study (ClinicalTrials.gov, NCT03473925). Patients were randomized 1:1 via an interactive response technology system to receive either navarixin 30 or 100 mg orally once daily in combination with pembrolizumab 200 mg intravenously every 3 weeks. The navarixin doses studied in this trial were selected based on the pharmacokinetic (PK)-ANC relationship observed in healthy volunteers from phase 1 studies.

Randomization was stratified according to tumor type. Treatment with combination therapy continued up to 35 administrations (~2 years) of pembrolizumab or until radiographic confirmation of disease progression, unacceptable toxicity, intercurrent illness preventing treatment administration, protocol noncompliance, or investigator’s decision to withdraw the patient.

### Endpoints

Primary endpoints were objective response rate (ORR; proportion of patients with confirmed complete or partial response) by RECIST v1.1 as assessed by the investigator and safety based on the occurrence of dose-limiting toxicities (DLTs), adverse events (AEs), and treatment discontinuations because of AEs. Secondary endpoints included investigator-assessed ORR by immune-related RECIST (iRECIST), investigator-assessed progression-free survival (PFS; time from first dose of study drug until the first documented disease progression or death from any cause) by RECIST v1.1 and iRECIST, overall survival (OS; time from first dose of study drug until death from any cause), pharmacodynamics (assessed via changes in ANC), and PK of navarixin when administered in combination with pembrolizumab.

### Assessments

Tumor imaging was performed at screening and every 9 weeks thereafter, at treatment discontinuation, and every 12 weeks after treatment discontinuation until the start of new anticancer therapy, disease progression, pregnancy, death, withdrawal of consent, or end of study, whichever occurred first. DLTs were defined as any nonhematologic toxicity (not including changes in laboratory values) of grade 4 or of grade 3 lasting >3 days despite optimal supportive care; grade 4 anemia of any duration or grade 3 anemia lasting >7 days or requiring transfusion; grade 4 hematologic toxicity (aside from anemia) lasting ≥7 days; grade 4 thrombocytopenia or grade 3 thrombocytopenia associated with bleeding; clinically significant, untreatable, or irreversible grade 3 or 4 nonhematologic laboratory values that required medical intervention, led to hospitalization, or persisted for >72 hours; alanine aminotransferase or aspartate aminotransferase >3-fold upper limit of normal (ULN) with total bilirubin >2-fold ULN and alkaline phosphatase <2-fold ULN, without other plausible explanations (eg, viral hepatitis); grade 3 or 4 febrile neutropenia; inability to administer ≥75% of planned navarixin dose because of drug-related toxicity; or a >2-week delay in starting cycle 2 because of toxicity. AEs were collected from randomization through 30 days after treatment discontinuation (90 days for serious AEs) and assessed using National Cancer Institute Common Terminology Criteria for Adverse Events version 4.03.

Blood samples for assessment of ANC were collected predose on day 1 of every cycle; between 6 and 12 hours after dosing on days 1, 3, and 8 of cycle 1; and between 6 and 12 hours after dosing on day 1 of cycle 2. Blood samples for assessment of navarixin pharmacokinetic parameters were collected predose and at 1, 2, 4, 6, and between 8 and 12 hours after dosing on day 1 of cycle 1; predose and between 6 and 12 hours after dosing on days 3 and 8 of cycle 1; predose and at 1, 2, 4, 6, and between 8 and 12 hours after dosing on day 1 of cycle 2; and predose during cycles 3 and 4.

### Statistical analysis

The threshold for an ORR to be considered clinically meaningful was ≥20% for CRPC, ≥16% for MSS CRC, and ≥20% for PD-(L)1–refractory NSCLC and was based upon 40 patients in each tumor type and 20 patients per tumor type assigned to 1 of 2 doses of navarixin (30 or 100 mg daily) under a 2-stage adaptive design. These historic benchmarks were selected based on prior studies of pembrolizumab monotherapy in these 3 settings [21–23]. The total sample size was therefore planned at 120 patients total (40 per tumor type with 20 in each treatment arm). No formal hypothesis testing was performed as each treatment group was compared against historic control data.

The efficacy population included all patients with measurable disease at baseline who received ≥1 dose of study drug. The safety population included all patients who received ≥1 dose of study drug. DLTs were assessed in all patients in the safety population who completed cycle 1 without a DLT or who experienced a DLT in cycle 1. The pharmacodynamic and PK populations included all patients who complied with the protocol sufficiently to ensure their data were likely to exhibit treatment effects. ORR was estimated using an exact method based on binomial distribution and 95% CI (Clopper-Pearson interval). PFS and OS were analyzed using the Kaplan-Meier method.

A protocol-specified interim safety analysis was to occur after the first 10 patients across tumor types completed ≥1 treatment cycle in a given treatment arm. If ≤3 of the 10 patients experienced a DLT during cycle 1, the treatment arm could be expanded up to 30 patients total per tumor type. An interim futility analysis was also planned for each tumor type and each treatment arm after the first 10 patients in each tumor type had ≥1 postbaseline assessment. The enrollment of patients was not paused for each interim futility analysis. If ≥1 response was observed in a tumor type (ie, ≥10% response rate), the tumor type could be expanded to enroll ≥10 additional patients in stage 2. Otherwise, that tumor type was stopped early due to futility.

## Results

### Patients

The study was conducted between December 18, 2018, and May 19, 2021. One-hundred five patients (navarixin 30-mg group, n=51; navarixin 100-mg group, n=54) were enrolled at 14 study sites in Australia, Canada, Israel, South Korea, and the United States. Patient disposition is summarized in Online Resource 1. At the database cutoff date of May 19, 2021, the median duration of follow-up was 10.1 (range, 1.1−35.9) months for the overall population. The median number of days on therapy was 63 (range, 14–744) for patients who received navarixin 30 mg and 64 (range, 4–772) for patients who received navarixin 100 mg. The median number of navarixin administrations was 63 (range, 14–737) in the 30-mg group and 64 (range, 4–733) in the 100-mg group; median number of pembrolizumab administrations was 3 (range, 1–35) in both navarixin groups. The study was closed based on the ORR and PFS results of the below prespecified interim futility analysis. No safety concerns were observed that required closing the study.

Patient demographics, baseline disease characteristics, and prior therapies were generally similar between treatment arms within each tumor type (Table 1). In the overall population, 74% of patients were men, median age was 64 years, and 66% of patients had an ECOG performance status of 1. The distribution of tumor types was 38% (40 of 105 patients) CRPC, 38% (40 of 105 patients) MSS CRC, and 24% (25 of 105 patients) NSCLC.

**Table 1:** Patient demographics and baseline disease characteristics in patients with **A**) castration-resistant prostate cancer, **B** microsatellite-stable colorectal cancer, and (**C**) non–small-cell lung cancer

**A. Castration-resistant prostate cancer**		
	**Navarixin 30 mg + Pembrolizumab 200 mg**	**Navarixin 100 mg + Pembrolizumab 200 mg**
	**n = 20**	**n = 20**
Age, median (range), y	74.5 (59–87)	71.0 (41–86)
<65, n (%)	4 (20)	7 (35)
≥65, n (%)	16 (80)	13 (65)
Sex, n (%)		
Male	20 (100)	20 (100)
Female	0	0
ECOG PS, n (%)		
0	4 (20)	8 (40)
1	16 (80)	12 (60)
No. of prior lines of therapy, n (%)		
1	1 (5)	0
2	3 (15)	3 (15)
3	6 (30)	4 (20)
4	5 (25)	8 (40)
≥5	5 (25)	5 (25)
Missing	0	0
Prior PD-(L)1 treatment history, n (%)		
Yes	1 (5)	0
No	19 (95)	20 (100)

**B. Microsatellite-stable colorectal cancer**		
	**Navarixin 30 mg + Pembrolizumab 200 mg**	**Navarixin 100 mg + Pembrolizumab 200 mg**
	**n = 19**	**n = 21**
Age, median (range), y	55 (36–77)	59 (30–76)
<65, n (%)	15 (79)	17 (81)
≥65, n (%)	4 (21)	4 (19)
Sex, n (%)		
Male	10 (53)	12 (57)
Female	9 (47)	9 (43)
ECOG PS, n (%)		
0	3 (16)	12 (57)
1	16 (84)	9 (43)
No. of prior lines of therapy, n (%)		
1	2 (11)	0
2	4 (21)	8 (38)
3	6 (32)	7 (33)
4	5 (26)	3 (14)
≥5	2 (11)	2 (10)
Missing	0	1 (5)
Prior PD-(L)1 treatment history, n (%)		
Yes	0	0
No	19 (100)	21 (100)

**C. Non–small-cell lung cancer**		
	**Navarixin 30 mg + Pembrolizumab 200 mg**	**Navarixin 100 mg + Pembrolizumab 200 mg**
	**n = 12**	**n = 13**
Age, median (range), y	63 (36–83)	71 (46–79)
<65, n (%)	6 (50)	4 (31)
≥65, n (%)	6 (50)	9 (69)
Sex, n (%)		
Male	7 (58)	9 (69)
Female	5 (42)	4 (31)
ECOG PS, n (%)		
0	4 (33)	5 (38)
1	8 (67)	8 (62)
No. of prior lines of therapy, n (%)^a^		
1	3 (25)	4 (31)
2	4 (33)	5 (38)
3	4 (33)	0
4	1 (8)	3 (23)
≥5	0	0
Missing	0	0
Prior PD-(L)1 treatment history, n (%)		
Yes	9 (75)	11 (85)
No	3 (25)	2 (15)

ECOG PS, Eastern Cooperative Oncology Group performance status; PD-L1, programmed cell death ligand 1

^a^One patient in the navarixin 100-mg arm received prior (neo)adjuvant therapy

### Efficacy

**Fig. 1:**
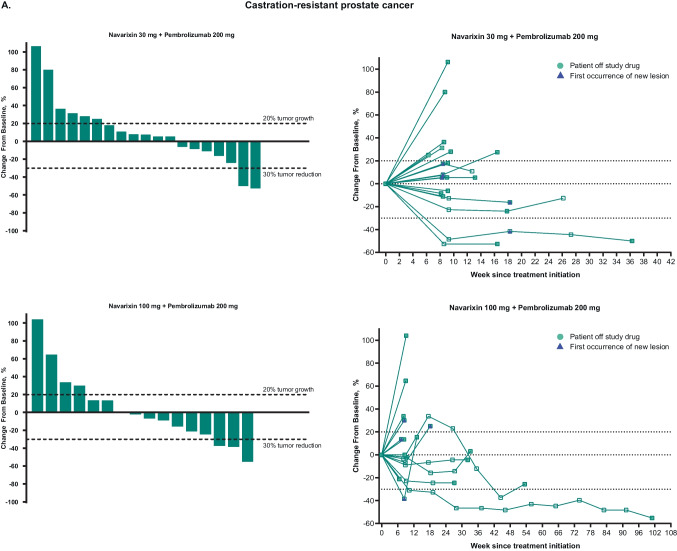

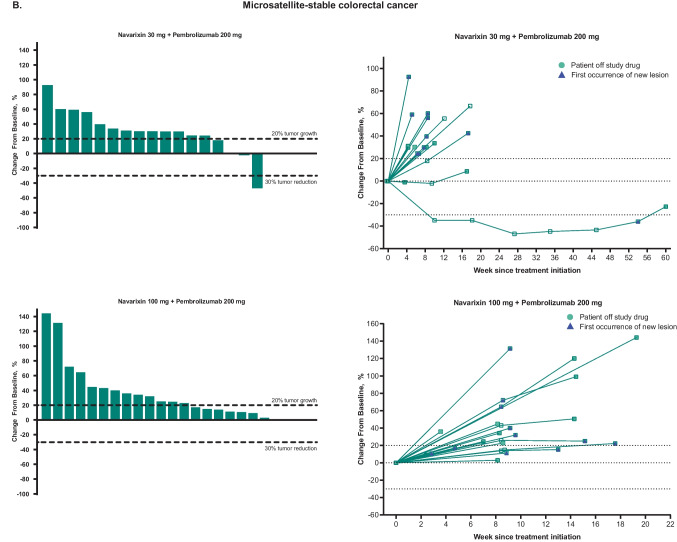

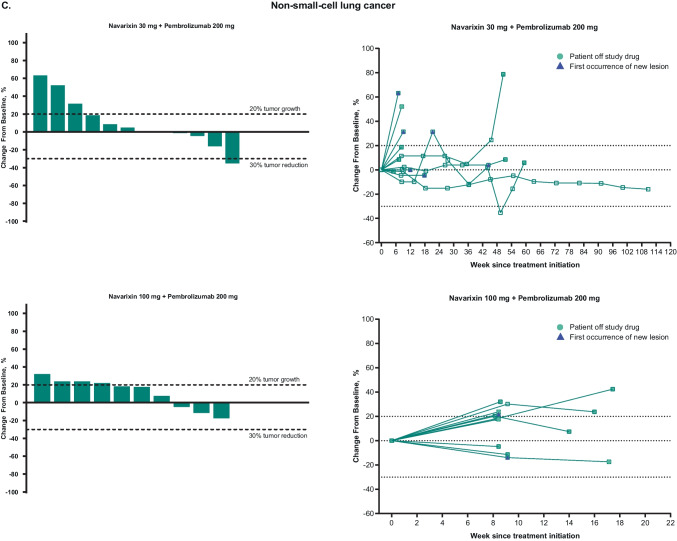
Maximum percentage change from baseline in target lesions and spider plots of percentage change from baseline for target lesions, based on investigator assessment per Response Evaluation Criteria in Solid Tumors version 1.1, in patients with **A** castration-resistant prostate cancer, **B** microsatellite-stable colorectal cancer, and **C** non–small-cell lung cancer. The last assessment before discontinuation of study drug (or within 1 cycle of discontinuation of study drug) is plotted as ‘Patient off study drug’ in the spider plots

No patient achieved a complete response (Table 2). The ORR in CRPC was 5% at each dose level and 5% for MSS CRC at 30 mg, but was 0% for all other cohorts, thus meeting the prespecified futility rules. Partial responses were attained by 2 patients treated with navarixin 30 mg (n=1, CRPC; n=1, MSS CRC) and 1 patient treated with navarixin 100 mg (CRPC). Thus, the overall ORR was 5% in men with CRPC, 2.5% in patients with MSS CRC, and 0% in patients with PD-(L)1–resistant NSCLC. There were no responses by iRECIST (Online Resource 2). A reduction of ≥30% in target lesion size was observed in 5 patients (13%) with CRPC, 1 patient (3%) with MSS CRC, and 1 patient (4%) with NSCLC (Fig. 1).

**Table 2:** Efficacy results

	**CRPC**		**MSS CRC**		**NSCLC**	
	**Navarixin 30 mg + Pembrolizumab 200 mg** **n = 20**	**Navarixin 100 mg + Pembrolizumab 200 mg** **n = 20**	**Navarixin 30 mg + Pembrolizumab 200 mg** **n = 19**	**Navarixin 100 mg + Pembrolizumab 200 mg** **n = 21**	**Navarixin 30 mg + Pembrolizumab 200 mg** **n = 12**	**Navarixin 100 mg + Pembrolizumab 200 mg** **n = 13**
Objective response rate, %^a^	5	5	5	0	0	0
Objective response rate for disease state, %^a^	5		2.5		0	
Best overall response, n (%)^a^						
Complete response	0	0	0	0	0	0
Partial response	1 (5)	1 (5)	1 (5)	0	0	0
Stable disease	6 (30)	6 (30)	2 (11)	3 (14)	6 (50)	3 (23)
Progressive disease	12 (60)	10 (50)	15 (79)	16 (76)	6 (50)	8 (62)
Nonevaluable^b^	0	0	0	1 (5)	0	0
No assessment^c^	1 (5)	3 (15)	1 (5)	1 (5)	0	2 (15)
PFS^a^						
Events, n (%)	19 (95)	17 (85)	19 (100)	21 (100)	11 (92)	12 (92)
Median (95% CI), mo	2.1 (1.9‒4.1)	2.1 (1.9‒4.5)	1.8 (1.0‒2.0)	1.9 (1.6‒2.0)	2.4 (1.6‒10.2)	2.1 (1.9‒2.4)
6-mo PFS rate (95% CI), %	16.2 (4.0‒35.5)	21.5 (6.7‒41.7)	10.5 (1.8‒28.4)	4.8 (0.3‒19.7)	25.0 (6.0‒50.5)	0 (NR‒NR)
OS						
Death, n (%)	18 (90)	15 (75)	19 (100)	20 (95)	11 (92)	9 (69)
Median (95% CI), mo	10.8 (7.9‒13.4)	11.2 (3.7‒23.0)	6.5 (3.0‒9.7)	8.0 (5.7‒14.4)	13.0 (3.2‒18.0)	12.0 (2.4‒19.9)
6-mo OS rate (95% CI), %	89.7 (64.8‒97.3)	55.0 (31.3‒73.5)	57.9 (33.2‒76.3)	70.8 (46.2‒85.7)	75.0 (40.8‒91.2)	73.3 (37.9‒90.6)

CRPC, castration-resistant prostate cancer; MSS CRC, microsatellite-stable colorectal cancer; NR, not reached; NSCLC, non–small-cell lung cancer; OS, overall survival; PFS, progression-free survival; RECIST, Response Evaluation Criteria in Solid Tumors

^a^Per RECIST version 1.1 as assessed by the investigator

^b^Includes patients with (a) no imaging/measurement done at all or only partial lesion measurements done at first time point and subsequent time point; (b) complete response at first time point but with no imaging/measurement done at all or only partial lesion measurements done without meeting the minimum criteria for stable disease duration at subsequent time point; (c) partial response at first time point but with no imaging/measurement done at all or only partial lesion measurements done without meeting the minimum criteria for stable disease duration at subsequent time point

^c^Includes patients without postbaseline assessment at the database cutoff date

In the navarixin 30-mg and 100-mg groups, median (95% CI) PFS per RECIST v1.1 was 2.1 (1.9–4.1) months and 2.1 (1.9–4.5) months, respectively, for patients with CRPC, 1.8 (1.0–2.0) months and 1.9 (1.6–2.0) months for patients with MSS CRC, and 2.4 (1.6–10.2) months and 2.1 (1.9–2.4) months for patients with NSCLC (Table 2, Online Resource 3**)**. Median (95% CI) OS was 10.8 (7.9–13.4) months and 11.2 (3.7–23.0) months for patients with CRPC, 6.5 (3.0–9.7) months and 8.0 (5.7–14.4) months for patients with MSS CRC, and 13.0 (3.2–18.0) months and 12.0 (2.4–19.9) months for patients with NSCLC (Table 2). Results for PFS by iRECIST are summarized in Online Resource 2. Outcomes did not differ within each pharmacodynamic dose level of navarixin for each of the selected tumor types.

### Safety

Dose-limiting toxicities were reported in 2 of 48 patients (4%) receiving navarixin 30 mg and 3 of 48 patients (6%) receiving navarixin 100 mg. Patients in the navarixin 30-mg group had DLTs of grade 4 decreased neutrophil count (lasting 2 days) and grade 3 increased transaminases (n=1 each). Patients in the navarixin 100-mg group had DLTs of grade 4 neutropenia (lasting ~2 months), grade 3 hepatitis, and grade 3 pneumonitis (n=1 each).

Seventy of 105 patients (67%) in the safety population experienced treatment-related AEs (Table 3). The most common were decreased neutrophil count (navarixin 30 mg, 14%; navarixin 100 mg, 17%), neutropenia (10% and 17%, respectively), fatigue (14% and 9%, respectively), and pruritus (12% and 11%, respectively). Seven patients (7%) discontinued study drug because of treatment-related AEs. One patient in the navarixin 30-mg group committed suicide; the patient had no history of psychiatric disorders, and the investigator could not rule out a relationship between study treatment and the patient’s death.

**Table 3:** Treatment-related adverse events

**Treatment-Related Adverse Event, n (%)**	**Navarixin 30 mg + Pembrolizumab 200 mg** **n = 51**		**Navarixin 100 mg + Pembrolizumab 200 mg** **n = 54**	
Any grade	37 (73)		33 (61)	
Grade 3−5	13 (25)		12 (22)	
Led to discontinuation of study drug(s)	3 (6)		4 (7)	
Led to death	1 (2)		0	

	**Any Grade**	**Grade 3–5**	**Any Grade**	**Grade 3–5**
Most common (≥2 patients in either treatment arm)^a^				
Neutrophil count decreased	7 (14)	5 (10)	9 (17)	4 (7)
Neutropenia	5 (10)	1 (2)	9 (17)	6 (11)
Fatigue	7 (14)	0	5 (9)	0
Pruritus	6 (12)	0	6 (11)	0
Cough	3 (6)	1 (2)	3 (6)	0
White blood cell count decreased	3 (6)	1 (2)	3 (6)	1 (2)
Pyrexia	2 (4)	0	3 (6)	0
Hypothyroidism	2 (4)	0	2 (4)	0
Nausea	1 (2)	0	3 (6)	0
Vomiting	1 (2)	0	3 (6)	0
Decreased appetite	3 (6)	1 (2)	0	0
Hyperthyroidism	2 (4)	0	1 (2)	0
Rash maculo-papular	2 (4)	0	1 (2)	0
Chills	0	0	2 (4)	0
Diarrhea	0	0	2 (4)	0
Rash	0	0	2 (4)	0

^a^Among treatment-related adverse events of any grade, pneumonitis was reported in 1 patient in each treatment arm and colitis was reported in 1 patient in the navarixin 30-mg arm; neutropenic fever was not reported as a treatment-related adverse event in either treatment arm

### Pharmacodynamics

No apparent differences were observed between the treatment arms in maximum mean percentage change from baseline in ANC (Online Resource 4). Maximum reductions from baseline in ANC were 44.5% to 48.2% during cycle 1 and 37.5% to 44.2% during cycle 2 and had occurred within 6 to 12 hours postdose in both treatment arms. ANC values returned to baseline faster with navarixin 30 mg than with navarixin 100 mg; predose reductions were 14.0% (lower dose) and 26.6% (higher dose) on day 1 of cycle 2 and 5.2% and 25.1%, respectively, on day 1 of cycle 3. Of all ANC measurements (multiple time points per patient), 3% and 7% of values were <1 × 10^9^/L in the navarixin 30-mg and 100-mg groups, respectively, and 0.4% and 0.7% of values were <0.5 × 10^9^/L, respectively.

### Pharmacokinetics

Within each treatment arm, no apparent differences were observed in navarixin PK parameters across tumor types on day 1 of cycle 1 and on day 1 of cycle 2 (steady state) (Table 4). At both time points, geometric mean area-under-the-curve (AUC) values and peak concentrations were higher with navarixin 100 mg than with navarixin 30 mg, regardless of tumor type. AUC_0-∞_ values, for example, were approximately 2.5-fold higher with navarixin 100 mg than with navarixin 30 mg across all tumor types. At steady state (all tumor types combined), geometric mean trough concentrations were 7.71 ng/mL with navarixin 30 mg and 14.5 ng/mL with navarixin 100 mg. Navarixin plasma concentration-time profiles are shown in Online Resource 5.

**Table 4:** Pharmacokinetic results

	**CRPC**		**MSS CRC**		**NSCLC**	
	**Navarixin 30 mg + Pembrolizumab 200 mg**	**Navarixin 100 mg + Pembrolizumab 200 mg**	**Navarixin 30 mg + Pembrolizumab 200 mg**	**Navarixin 100 mg + Pembrolizumab 200 mg**	**Navarixin 30 mg + Pembrolizumab 200 mg**	**Navarixin 100 mg + Pembrolizumab 200 mg**
Cycle 1 day 1						
n	20	20	19	21	12	13
AUC_0–inf_, h∙ng/mL	376 (43.8)^a^	954 (50.8)^b^	482 (36.5)^c^	1120 (36.0)^d^	469 (47.5)^e^	1230 (46.3)^f^
AUC_0–last_, h∙ng/mL	363 (46.0)	853 (50.0)	464 (36.6)	1070 (46.6)	427 (46.5)	1140 (57.8)
C_max_, ng/mL	156 (43.8)	298 (68.3)	162 (56.0)	354 (41.6)	181 (74.0)	324 (52.2)
t_last_, h^g^	8.00 (7.83–10.00)	8.00 (7.37–8.08)	8.0 (7.27–8.33)	8.00 (7.12–10.02)	8.00 (7.83–8.25)	8.00 (6.00–8.07)
Cycle 2 day 1						
n	18	17	15	18	12	11
AUC_0–inf_, h∙ng/mL	534 (49.1)	1340 (39.7)^h^	584 (24.4)^e^	954 (38.9)^f^	450 (30.6)^e^	1360 (62.4)^i^
AUC_0–last_, h∙ng/mL	457 (31.5)	925 (35.7)	443 (43.2)	928 (48.9)	451 (35.1)	1080 (57.4)
C_max_, ng/mL	191 (40.8)	272 (69.1)	147 (69.0)	276 (53.8)	179 (48.9)	338 (60.8)
t_last_, h^g^	8.01 (6.50–8.70)	8.00 (5.93–8.17)	7.93 (6.00–8.17)	8.00 (7.87–8.13)	8.00 (7.80–8.08)	8.00 (6.03–8.08)

Except where noted, data are geometric mean (% geometric coefficient of variation)

AUC_0–inf_, area under the concentration-time curve from time 0 through infinity; AUC_0–last_, area under the concentration-time curve from time 0 through last measurable concentration; C_max_, maximum concentration; CRPC, castration-resistant prostate cancer; MSS CRC, microsatellite-stable colorectal cancer; NSCLC, non–small-cell lung cancer; t_last_, time of last measurable concentration

^a^n = 19

^b^n = 18

^c^n = 17

^d^n = 16

^e^n = 10

^f^n = 9

^g^Data are median (range)

^h^n = 11

^i^n = 7

## Discussion

In this phase 2 study of the combination of navarixin 30 or 100 mg plus pembrolizumab 200 mg in patients with previously treated CRPC, MSS CRC, and NSCLC, there were no complete responses and 3 partial responses (n=2, CRPC; n=1, MSS CRC) for an overall ORR of 3%. We observed ORRs ranging from 0% to 5% across disease subtypes and dosing cohorts, and median PFS times were 1.8 to 2.4 months with modest OS results observed despite many patients achieving reduction in peripheral ANC by navarixin. There was no evidence of a dose-response relationship. The study was closed for lack of efficacy based on the results of the prespecified interim futility analysis. Navarixin plus pembrolizumab had manageable safety and tolerability, with no new safety signals detected and no increase in the risk of neutropenic fever or infections.

Although caution must be taken in comparing studies, navarixin plus pembrolizumab was associated with a similar or lower ORR compared with previous studies of pembrolizumab monotherapy in these solid tumor populations, irrespective of the navarixin dose level. In the phase 2 KEYNOTE-199 study, the ORR among patients with treatment-refractory (including docetaxel and endocrine therapy) metastatic CRPC was 5% (9/199) with pembrolizumab monotherapy [22]. Le et al reported an ORR of 0% in a phase 2 study that included 18 patients with mismatch repair–proficient CRC [21]. In the phase 1 KEYNOTE-001 study, the ORR in the previously treated advanced NSCLC cohort, half of whom had received ≥3 prior systemic therapies, was 23% (103/449) with pembrolizumab monotherapy [24]. As mentioned previously, increased CXCR2 expression in tumor cells and myeloid cells is responsible for recruitment of MDSC to the tumor microenvironment, which promotes tumor progression. The reduction in ANC observed after administration of navarixin was used as an easily monitorable functional biomarker and assumed to be a surrogate for target engagement based on the effect of navarixin in inhibiting the migration of neutrophils (neutrophil trafficking) to the blood [25, 26]. A recent study demonstrated a significant positive association between neutrophil count and tumor MDSC infiltration in patients with metastatic CRPC after treatment with another CXCR2 antagonist [27]. We speculate that the lack of efficacy in our study may be related to the modest ANC reductions (few of which were <0.5 or <1 × 10^9^/L), which may have been insufficient to overcome MDSC activity in the tumor microenvironment and tumor resistance to anti–PD-(L)1 treatment. Navarixin steady-state exposure following the 100-mg dose was approximately 2.5-fold higher compared with the 30-mg dose. However, even with such a wide exposure margin between the 2 navarixin doses, maximum ANC reductions were comparable with navarixin 30 mg and 100 mg and indicative of a flat exposure response. Moreover, most patients (90% at 30 mg, 83% at 100 mg) did not experience neutropenia as a treatment-related AE. As such, it is not likely that a higher dose of navarixin would have achieved a greater ANC reduction in this patient population, although we cannot rule out that suboptimal dosing and pharmacodynamic effects of navarixin may have limited the observed efficacy. The requirement for patients with NSCLC to have progressed on previous anti–PD-(L)1 treatment also may have influenced efficacy outcomes in that cohort. Alternatively, taken together with the results from studies with other CXCR1/2 antagonists, as described below, it is also possible that inhibition of CXCR1/2 is not an effective therapeutic approach in solid tumors as monotherapy or in combination with PD-1 blockade.

Other CXCR2 or CXCR1/2 antagonists have been investigated in patients with solid tumors. The phase 1/2 SCORES study assessed either CXCR2 antagonist AZD5069 or the STAT3 inhibitor AZD9150 in combination with the PD-L1 inhibitor durvalumab [28, 29]. Initial data from the study suggested that the combinations were safe and tolerable. Complete responses were reported in 2 patients (breast cancer, prostate cancer), and partial responses occurred in multiple unspecified tumor types. Sample sizes were not provided and it was unclear whether the responses were associated with CXCR2 and/or STAT3 inhibition. In the dose-expansion phase of SCORES, which only included patients with recurrent or metastatic squamous cell carcinoma of the head and neck, ORRs were 5% among 22 previously untreated patients and 10% among the 20 patients who previously received a PD-L1 inhibitor [28, 29]. A phase 2 study also evaluated the combination of AZD5069 plus durvalumab in patients with metastatic pancreatic ductal adenocarcinoma, with 6% of 18 patients achieving an objective response [30]. An ongoing phase 1/2 study (NCT03177187) is assessing the combination of AZD5069 plus the androgen receptor antagonist enzalutamide in patients with metastatic CRPC who have progressed on prior enzalutamide [31]. In this study, induced neutropenia was observed in the majority of patients, and 3 of 21 patients achieved a partial response [32], despite the observation of major drug interactions with enzalutamide limiting the levels of AZD5069. The only other studies of CXCR2 or CXCR1/2 antagonists in patients with solid tumors that we are aware of involve the small-molecule CXCR1/2 inhibitor reparixin, administered either as monotherapy [33] or in combination with paclitaxel [34, 35] in patients with human epidermal growth factor receptor 2‒negative breast cancer. In the only study with efficacy as a primary objective, no benefit was seen with combination therapy compared with paclitaxel alone in patients with metastatic triple-negative breast cancer [35].

A limitation of our study was that we did not collect tumor biopsies. Another limitation was the lack of titration of dosing to inhibition of MDSC activity in the peripheral blood or in tumor biopsies and the subsequent lack of efficacy observed at higher navarixin doses, which in most patients did not achieve the desired degree of neutrophil reduction. This may not have been achievable with navarixin due to its more limited bioavailability, given the lack of a dose response or exposure response observed. Future studies of CXCR2 or MDSC inhibitors should carefully consider the optimal pharmacodynamic dose level for combination studies. In addition, in metastatic CRPC, reversal of lineage plasticity may require combination approaches with androgen receptor blockade to achieve greater efficacy [6].

In our study of patients with advanced or metastatic CRPC, MSS CRC, and PD-(L)1–refractory NSCLC, the ORR associated with the combination of navarixin plus pembrolizumab was lower than that previously reported in patients treated with pembrolizumab monotherapy. Although there was evidence of pharmacodynamic activity of navarixin, the observed reductions in ANC were modest. The study was closed for lack of efficacy after an interim futility analysis. Safety and tolerability of the combination were manageable. Our findings may inform future studies of CXCR2 antagonists in combination with anti–PD-(L)1 treatments.

### Supplementary Information

Below is the link to the electronic supplementary material.

Supplementary file1 (DOCX 748 KB)

## Data Availability

Merck Sharp & Dohme LLC, a subsidiary of Merck & Co., Inc., Rahway, NJ, USA (MSD) is committed to providing qualified scientific researchers access to anonymized data and clinical study reports from the company’s clinical trials for the purpose of conducting legitimate scientific research. MSD is also obligated to protect the rights and privacy of trial participants and, as such, has a procedure in place for evaluating and fulfilling requests for sharing company clinical trial data with qualified external scientific researchers. The MSD data sharing website (available at: http://engagezone.msd.com/ds_documentation.php) outlines the process and requirements for submitting a data request. Applications will be promptly assessed for completeness and policy compliance. Feasible requests will be reviewed by a committee of MSD subject matter experts to assess the scientific validity of the request and the qualifications of the requestors. In line with data privacy legislation, submitters of approved requests must enter into a standard data-sharing agreement with MSD before data access is granted. Data will be made available for request after product approval in the US and EU or after product development is discontinued. There are circumstances that may prevent MSD from sharing requested data, including country or region-specific regulations. If the request is declined, it will be communicated to the investigator. Access to genetic or exploratory biomarker data requires a detailed, hypothesis-driven statistical analysis plan that is collaboratively developed by the requestor and MSD subject matter experts; after approval of the statistical analysis plan and execution of a data-sharing agreement, MSD will either perform the proposed analyses and share the results with the requestor or will construct biomarker covariates and add them to a file with clinical data that is uploaded to an analysis portal so that the requestor can perform the proposed analyses.
