# Correction to: CXCR2 antagonist navarixin in combination with pembrolizumab in select advanced solid tumors: a phase 2 randomized trial

**DOI:** 10.1007/s10637-024-01444-0

**Published:** 2024-05-20

**Authors:** Andrew J. Armstrong, Ravit Geva, Hyun Cheol Chung, Charlotte Lemech, Wilson H. Miller, Aaron R. Hansen, Jong-Seok Lee, Frank Tsai, Benjamin J. Solomon, Tae Min Kim, Christian Rolfo, Vincent Giranda, Yixin Ren, Fang Liu, Bhargava Kandala, Tomoko Freshwater, Judy S. Wang

**Affiliations:** 1 Duke Cancer Institute Center for Prostate and Urologic Cancers, Duke University, Durham, NC 27710 USA; 2 Division of Oncology, Tel Aviv Sourasky Medical Center, Tel Aviv, Israel, affiliated to the Sackler School of Medicine, Tel Aviv University, Tel Aviv, Israel; 3 Yonsei Cancer Center, Yonsei University Health System, Seoul, South Korea; 4 Scientia Clinical Research, Randwick, NSW Australia; 5 Segal Cancer Center, McGill University, Jewish General Hospital, Montreal, QC Canada; 6 Princess Margaret Cancer Centre, Toronto, ON Canada; 7 Seoul National University Bundang Hospital, Gyeonggi-Do, South Korea; 8 Honor Health, Scottsdale, AZ USA; 9 Peter MacCallum Cancer Centre, Melbourne, VIC Australia; 10 Seoul National University Hospital, Seoul, South Korea; 11 Center for Thoracic Oncology, Icahn School of Medicine at Mount Sinai, The Tisch Cancer Institute, New York, NY USA; 12 Merck & Co., Inc, Rahway, NJ USA; 13 Florida Cancer Specialists/Sarah Cannon Research Institute, Sarasota, FL USA

**Correction to: Investigational New Drugs (2024) 42:145-159** 10.1007/s10637-023-01410-2

The article CXCR2 antagonist navarixin in combination with pembrolizumab in select advanced solid tumors: a phase 2 randomized trial, written by Andrew J. Armstrong, Ravit Geva, Hyun Cheol Chung, Charlotte Lemech, Wilson H. Miller Jr., Aaron R. Hansen, Jong‑Seok Lee, Frank Tsai, Benjamin J. Solomon, Tae Min Kim, Christian Rolfo, Vincent Giranda, Yixin Ren, Fang Liu, Bhargava Kandala, Tomoko Freshwater, Judy S. Wang, was originally published electronically on the publisher’s internet portal on February 7, 2024 without open access. With the author(s)’ decision to opt for Open Choice the copyright of the article changed on March 24, 2024 to ©The Author(s) 2024 and the article is forthwith distributed under a Creative Commons Attribution 4.0 International License, which permits use, sharing, adaptation, distribution and reproduction in any medium or format, as long as you give appropriate credit to the original author(s) and the source, were made. The images or other third party material in this article are included in the article's Creative Commons licence, unless indicated otherwise in a credit line to the material. If material is not included in the article's Creative Commons licence and your intended use is not permitted by statutory regulation or exceeds the permitted use, you will need to obtain permission directly from the copyright holder. To view a copy of this licence, visit http://creativecommons.org/licenses/by/4.0/. Merck & Co., Inc., Rahway, NJ, USA and its affiliates and Andrew J. Armstrong, Ravit Geva, Hyun Cheol Chung, Charlotte Lemech, Wilson H. Miller Jr., Aaron R. Hansen, Jong-Seok Lee, Frank Tsai, Benjamin J. Solomon, Tae Min Kim, Christian Rolfo, Judy S. Wang 2024.

