# Supplementary material for: CXCR2 antagonist navarixin in combination with pembrolizumab in select advanced solid tumors: a phase 2 randomized trial
Source: Invest New Drugs. 2024 Feb 7;42(1):145–59. doi: 10.1007/s10637-023-01410-2 (PMC11076327; doi:10.1007/s10637-023-01410-2)
Supplement: Supplementary file 1 — Supplementary file1 (DOCX 748 KB) [file 10637_2023_1410_MOESM1_ESM.docx]

**CXCR2 Antagonist Navarixin in Combination With Pembrolizumab in Select Advanced Solid Tumors: A Phase 2 Randomized Trial**

***Investigational New Drugs***

Andrew J. Armstrong, Ravit Geva, Hyun Cheol Chung, Charlotte Lemech, Wilson H. Miller Jr., Aaron R. Hansen, Jong-Seok Lee, Frank Tsai, Benjamin J. Solomon, Tae Min Kim, Christian Rolfo, Vincent Giranda, Yixin Ren, Fang Liu, Bhargava Kandala, Tomoko Freshwater, Judy S. Wang

Corresponding author: Andrew J. Armstrong
Duke Cancer Institute Center for Prostate and Urologic Cancers
Duke University

20 Duke Medicine Circle, Box 103861

Durham, NC, 27710

Email: andrew.armstrong@duke.edu

# SUPPLEMENTARY INFORMATION

**Online Resource 1.** Patient disposition


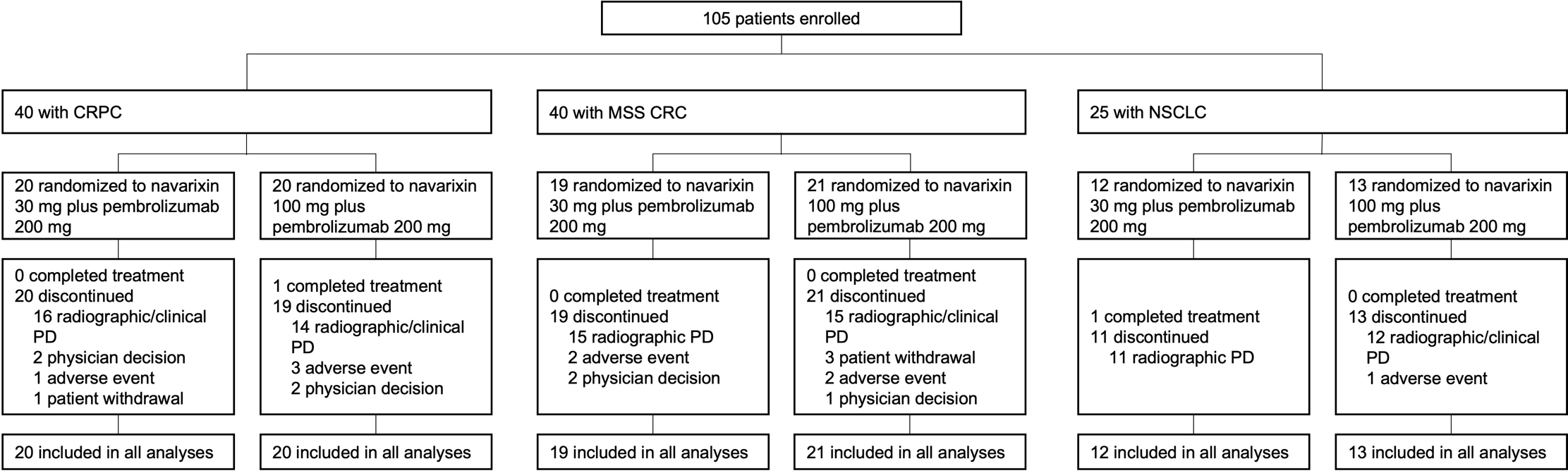


CRPC, castration-resistant prostate cancer; MSS CRC, microsatellite-stable colorectal cancer; NSCLC, non–small-cell lung cancer; PD, progressive disease.

**Online Resource 2.** Objective Response Rate (ORR) and Progression-Free Survival (PFS) by iRECIST

|  | **CRPC** | | **MSS CRC** | | **NSCLC** | |
| --- | --- | --- | --- | --- | --- | --- |
|  | **Navarixin 30 mg + Pembrolizumab 200 mg**  **n = 20** | **Navarixin 100 mg + Pembrolizumab 200 mg**  **n = 20** | **Navarixin 30 mg + Pembrolizumab 200 mg**  **n = 19** | **Navarixin 100 mg + Pembrolizumab 200 mg**  **n = 21** | **Navarixin 30 mg + Pembrolizumab 200 mg**  **n = 12** | **Navarixin 100 mg + Pembrolizumab 200 mg**  **n = 13** |
| Response by iRECIST^a^ |  |  |  |  |  |  |
| Patients in population, n | 15 | 14 | 17 | 18 | 11 | 9 |
| Best overall response, n (%)^a^ |  |  |  |  |  |  |
| Immune complete response | 0 | 0 | 0 | 0 | 0 | 0 |
| Immune partial response | 0 | 0 | 0 | 0 | 0 | 0 |
| Immune stable disease | 0 | 1 (7) | 0 | 0 | 1 (9) | 0 |
| Unconfirmed progressive disease | 10 (67) | 11 (79) | 13 (76) | 13 (72) | 8 (73) | 7 (78) |
| Confirmed progressive disease | 5 (33) | 2 (14) | 4 (24) | 5 (28) | 2 (18) | 2 (22) |
| PFS by iRECIST^a^ |  |  |  |  |  |  |
| Events, n (%) | 18 (90) | 16 (80) | 19 (100) | 20 (95) | 11 (92) | 9 (69) |
| Median (95% CI), mo | 7.9 (5.0, 8.6) | 5.2 (3.0, 11.5) | 4.1 (2.1, 7.1) | 6.2 (3.3, 14.4) | 11.9 (3.2, 17.5) | 7.0 (2.4, 14.4) |
| 6-mo PFS rate (95% CI), % | 72.6 (46.2, 87.6) | 46.0 (22.8, 66.5) | 42.1 (20.4, 62.5) | 50.4 (27.5, 69.5) | 75.0 (40.8, 91.2) | 50.5 (18.7, 75.7) |

CRPC, castration-resistant prostate cancer; iRECIST, immune-related Response Evaluation Criteria in Solid Tumors; MSS CRC, microsatellite-stable colorectal cancer; NSCLC, non–small-cell lung cancer.

^a^iRECIST was only assessed after progressive disease per RECIST v1.1. Therefore, only patients with progressive disease per RECIST v1.1 were included in iRECIST analyses.

**Online Resource 3**. Progression-free survival based on RECIST version 1.1 per investigator assessment in patients with (A) castration-resistant prostate cancer, (B) microsatellite-stable colorectal cancer, and (C) non–small-cell lung cancer

**A**.

**B**.

**C**.

**Online Resource 4.** Mean Absolute Neutrophil Count and Percentage Change From Baseline

|  | **Navarixin 30 mg + Pembrolizumab 200 mg** | | | **Navarixin 100 mg + Pembrolizumab 200 mg** | | |
| --- | --- | --- | --- | --- | --- | --- |
|  | **N** | **Mean (95% CI), 10^9^/L** | **Mean Change From Baseline (95% CI), %** | **N** | **Mean (95% CI, 10^9^/L)** | **Mean Change From Baseline (95% CI), %** |
| Baseline | 51 | 5.2 (4.50, 5.82) |  | 54 | 5.0 (4.38, 5.66) |  |
| Cycle 1, Day 1 |  |  |  |  |  |  |
| Predose | 51 | 4.6 (4.09, 5.20) |  | 54 | 4.9 (4.32, 5.57) |  |
| 6−12 h postdose | 48 | 2.4 (2.06, 2.81) | -48.2 (-51.83, -44.61) | 51 | 2.7 (2.25, 3.07) | -45.2 (-48.90, -41.54) |
| Cycle 1, Day 3 |  |  |  |  |  |  |
| Predose | 50 | 3.6 (3.03, 4.12) | -20.8 (-28.79, -12.73) | 51 | 3.2 (2.50, 3.84) | -37.3 (-46.00, -28.69) |
| 6−12 h postdose | 46 | 2.5 (2.08, 2.83) | -47.5 (-53.02, -42.02) | 52 | 2.5 (2.00, 3.09) | -48.2 (-56.30, -40.10) |
| Cycle 1, Day 8 |  |  |  |  |  |  |
| Predose | 50 | 3.7 (3.10, 4.30) | -17.2 (-28.11, -6.25) | 49 | 3.5 (2.79, 4.30) | -28.2 (-39.31, -17.18) |
| 6−12 h postdose | 46 | 2.6 (2.10, 3.12) | -44.5 (-51.75, -37.31) | 50 | 2.5 (2.04, 3.04) | -46.9 (-55.08, -38.76) |
| Cycle 2, Day 1 |  |  |  |  |  |  |
| Predose | 45 | 3.9 (3.13, 4.62) | -14.0 (-27.15, -0.84) | 46 | 3.4 (2.74, 4.10) | -26.6 (-37.29, -15.86) |
| 6−12 h postdose | 44 | 2.8 (2.26, 3.33) | -37.5 (-47.96, -26.96) | 46 | 2.7 (2.12, 3.27) | -44.2 (-51.52, -36.80) |
| Cycle 3, Day 1 |  |  |  |  |  |  |
| Predose | 36 | 4.4 (3.33, 5.44) | -5.2 (-19.83, 9.44) | 37 | 3.5 (2.72, 4.34) | -25.1 (-38.66, -11.48) |
| Cycle 4, Day 1 |  |  |  |  |  |  |
| Predose | 19 | 4.5 (3.31, 5.70) | 1.4 (-19.53, 22.42) | 23 | 3.8 (2.83, 4.78) | -8.3 (-38.70, 22.01) |
| Cycle 5, Day 1 |  |  |  |  |  |  |
| Predose | 15 | 4.3 (2.79, 5.79) | -8.7 (-33.29, 15.89) | 17 | 4.4 (2.87, 5.89) | -9.4 (-34.80, 15.96) |
| Cycle 6, Day 1 |  |  |  |  |  |  |
| Predose | 12 | 4.5 (2.94, 6.05) | -1.9 (-33.03, 29.17) | 8 | 3.0 (1.35, 4.74) | -41.4 (-61.26, -21.59) |

Data are not shown after cycle 6 due to sample size (n = <10 in both treatment groups).

**Online Resource 5.** Plasma concentration-time profiles of navarixin on day 1 of cycle 1 and day 1 of cycle 2 (steady state). CRPC, castration-resistant prostate cancer; MSS CRC, microsatellite-stable colorectal cancer; NSCLC, non–small-cell lung cancer

**A. Navarixin 30 mg on day 1 of cycle 1**


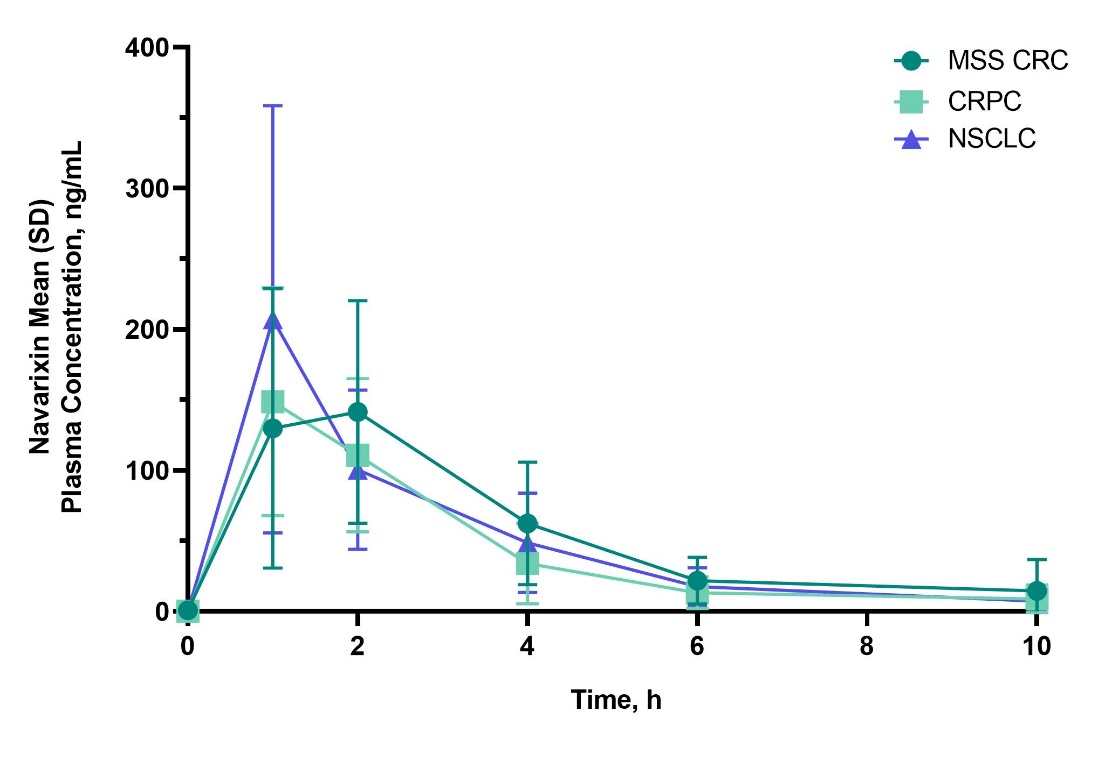


**B. Navarixin 30 mg on day 1 of cycle 2 (steady state)**

**
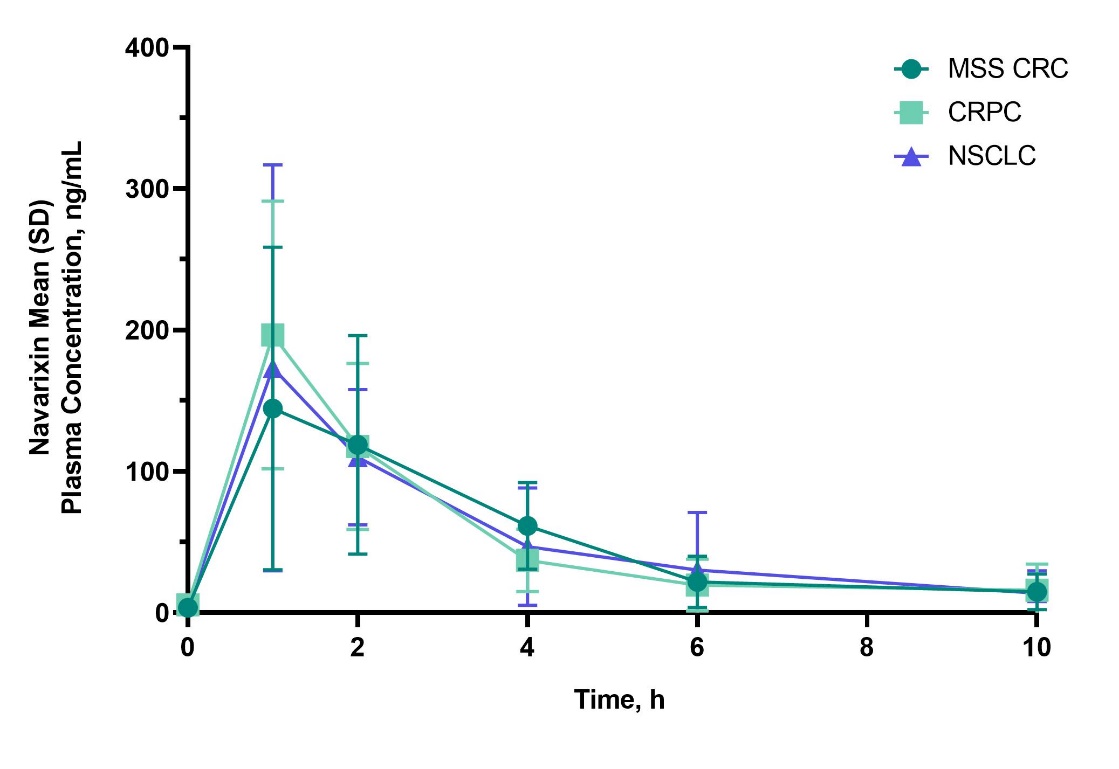
**

**C.** **Navarixin 100 mg on day 1 of cycle 1**

**
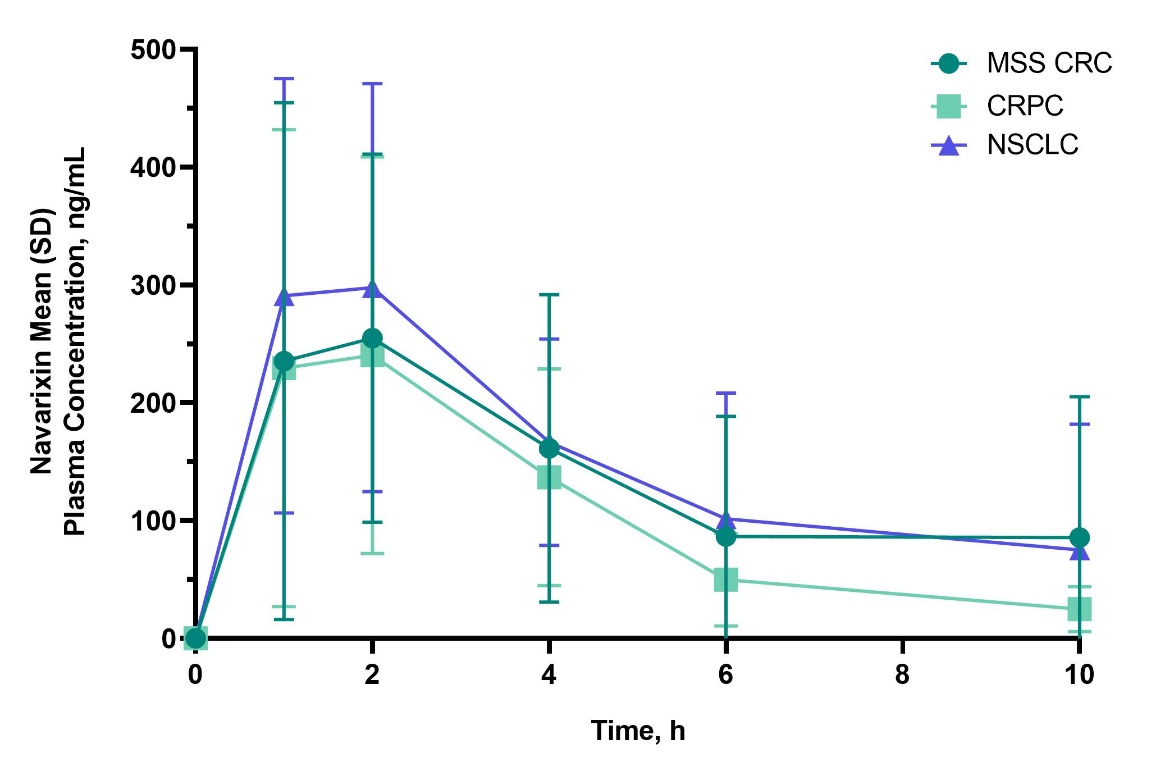
**

**D.** **Navarixin 100 mg on day 1 of cycle 2 (steady state)**

**
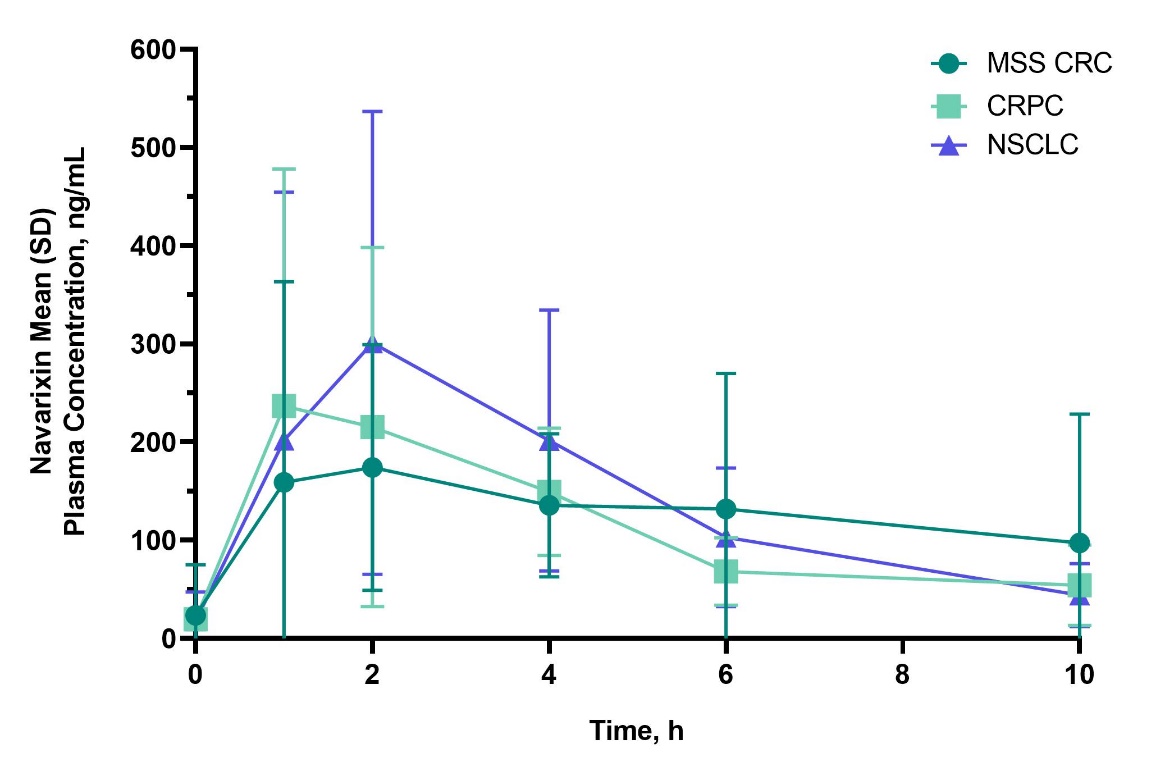
**
